# Complex periprosthetic wound coverage in patients undergoing revision total knee arthroplasty: a single plastic surgeon study

**DOI:** 10.1007/s00402-024-05240-6

**Published:** 2024-04-25

**Authors:** Marco Brenneis, Dimitrios A. Flevas, Lloyd B. Gayle, Friedrich Boettner, Peter K. Sculco, Geoffrey H. Westrich

**Affiliations:** 1 Stavros Niarchos Foundation Complex Joint Reconstruction Center, Hospital for Special Surgery, New York, NY USA; 2 Adult Reconstruction and Joint Replacement Service, Hospital for Special Surgery, New York, NY USA; 3 Division of Plastic Surgery, New York-Presbyterian Hospital, New York, NY USA; 4 Department of Orthopedics (Friedrichsheim), University Hospital Frankfurt, Goethe University, Frankfurt / Main, Germany

**Keywords:** Total knee arthroplasty, Complex wound closure, Gastrocnemius flap, Fasciocutaneous flap

## Abstract

**Introduction:**

Options for soft tissue coverage in revision total knee arthroplasty (rTKA) range from primary wound closure to complex muscle flap reconstructions. The purpose of this study was to investigate the institutional experience of wound coverage options for complex soft tissue defects in rTKA.

**Materials and methods:**

77 patients undergoing rTKA with complex wound closure by a single plastic surgeon were retrospectively reviewed. The average follow-up was 30.1 months. In 18 (23.4%) patients, an intraoperative decision for primary closure was made. Fifty-nine patients (76.6%) received either a local fasciocutaneous (*N* = 18), a medial gastrocnemius (*N* = 37), a free latissimus dorsi (*N* = 3) or a lateral gastrocnemius flap (*N* = 1). Revision-free survival and complication rates were assessed and risk factors were analyzed with Cox-regression analysis.

**Results:**

Medial gastrocnemius flaps had significant lower cumulative revision-free survival rates than local fasciocutaneous flaps (*P* = 0.021) and primary closures (*P* < 0.001) (42.5% vs. 71.5% vs. 100%,respectively). Comparing the most common complex closure procedures medial gastrocnemius flaps had the highest rate of prolonged wound healing (29.7%) and infection/reinfection (40.5%). Infection-associated flap procedures had significant lower cumulative revision-free survival rates (30.5%) than non-infection associated flap procedures (62.8%,*P* = 0.047). A history of more than two prior surgeries (HR = 6.11,*P* < 0.001) and an age ≥ 65 years (HR = 0.30,*P* = 0.018) significantly increased the risk of revision.

**Conclusions:**

The results of this study indicate that primary closure -if possible- should be preferred to early proactive muscle flap coverage. Even in the hands of an experienced plastic surgeon muscle flaps have high revision and complication rates. The study highlights the need to clarify flap indications and to investigate alternative approaches.

**Supplementary Information:**

The online version contains supplementary material available at 10.1007/s00402-024-05240-6.

## Introduction

The number of revision total knee arthroplasties (rTKA) has increased in recent years and is projected to increase between 78% and 182% by 2030 [1]. Postoperative wound complications are more common in patients suffering from diabetes, peripheral vascular disease or rheumatoid arthritis. Nicotine abuse, steroid use, subcutaneous positioning of the implants and an increased number of previous surgeries are additional risk factors for postoperative wound complications [2–4]. Once a complication has occurred, compromised wound healing due to multiple reoperations and associated devascularization of the local soft tissues can lead to catastrophic outcomes including loss of the implant, arthrodesis of the knee as well as amputation of the lower limb [2]. Consequently, an adequate soft-tissue coverage is essential.

Options for complex wound closures after total knee arthroplasties (TKA) range from primary wound closure to complex flap reconstructions often in combination with skin grafting. Recently, the fasciocutaneous flaps for reconstruction of small full-thickness soft tissue defects have gained popularity [5]. The preferred method for coverage of complex medium sized soft tissue defects is the medial gastrocnemius flap [6]. The advantages of this technique include the capacity to fill medium to large dead spaces, its robust blood supply, as well as the possibility to use an extension of the standard TKA approach for harvesting of the flap [7]. Nevertheless, for very large defects and defects extending superior to the patella, latissimus dorsi free flaps are often preferred [8].

Previous research comparing different defect coverage options is inconsistent due to their small case numbers and high case variability. The purpose of the current study is to investigate the outcome of soft tissue reconstructions and muscle flaps for complex wound defects in patients with TKA. Therefore, we asked the following research questions:

1. What is the cumulative revision free survival rate for different complex periprosthetic wound coverage procedures in patients undergoing rTKA?
2. What complications and complication rates should be expected after different periprosthetic wound coverage procedures?
3. What are the risk factors of reoperation after different periprosthetic wound coverage procedures?

## Methods

This single center retrospective study was approved by the Institutional Review Board (IRB number 2022 − 0964). In total, 77 consecutive patients (mean follow-up, 30.2 [range 0.4–112.5] months) who underwent rTKA with complex soft tissue coverage by a single plastic surgeon between 2012 and 2022 were retrospectively reviewed. Patients with a follow-up of less than 6 months were excluded, except if they experienced any complication before that period of time.

In cases with complicated wound conditions (characterized by the presence of foreign materials and factors including infection, tissue breakdown or poor blood supply), a, highly experienced plastic surgeon (LG) (> 50 muscle flaps/year) was consulted prior to surgery and the wound closure was subsequently performed by him. All patients had potential skin compromise due to multiple prior incisions, significant scarring, soft tissue compromise from extensive prior debridements, or open wounds. In 18 (23.4%) patients, an intraoperative decision for primary closure was made by the same plastic surgeon. The remaining 59 patients (76.6%) received either a local fasciocutaneous flap (*N* = 18), a medial gastrocnemius flap (*N* = 37), a free latissimus dorsi flap (*N* = 3) or a lateral gastrocnemius flap (*N* = 1).

**Fig. 1:**
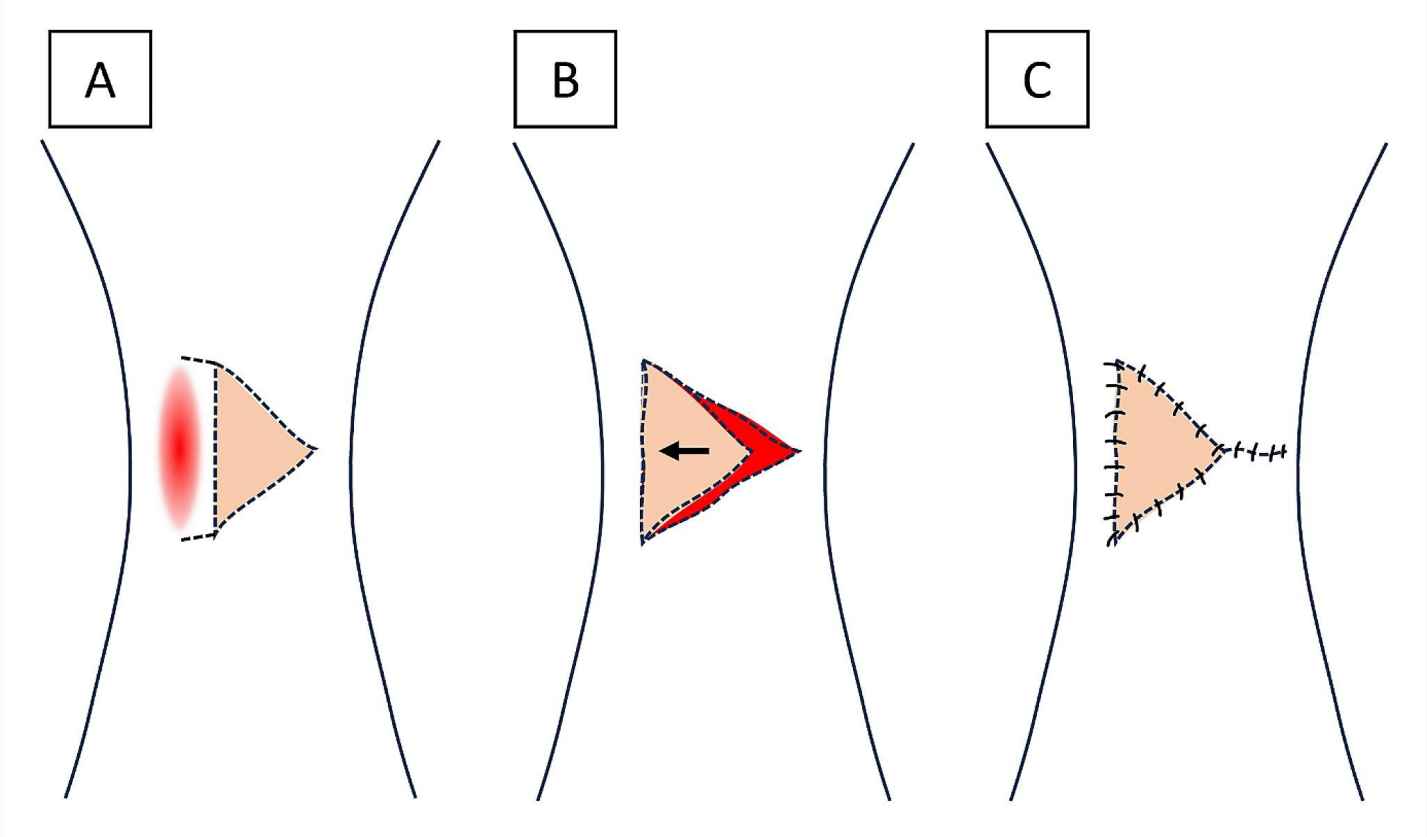
Technique for a V–Y fasciocutaneous flap. **(A)** Initial incision. **(B)** Mobilization of the flap to cover the defect. **(C)** Closure

When it was possible to primarily close the defect, the preferred technique was multilayer primary wound closure without flap coverage. In cases with small size full-thickness soft tissue defects, a local fasciocutaneous flap was utilized. This procedure involves the transfer of a flap of skin and underlying tissue from an adjacent area of the knee to cover the defect. First, an incision along the borders of the flap is made, carefully preserving the blood supply of the flap. The incision is made in a way that minimizes tension on the flap when it is moved to cover the defect. Afterwards, the fasciocutaneous flap is elevated of the surrounding tissue and transferred or rotated to cover the wound or tissue defect (Fig. 1).

**Fig. 2:**
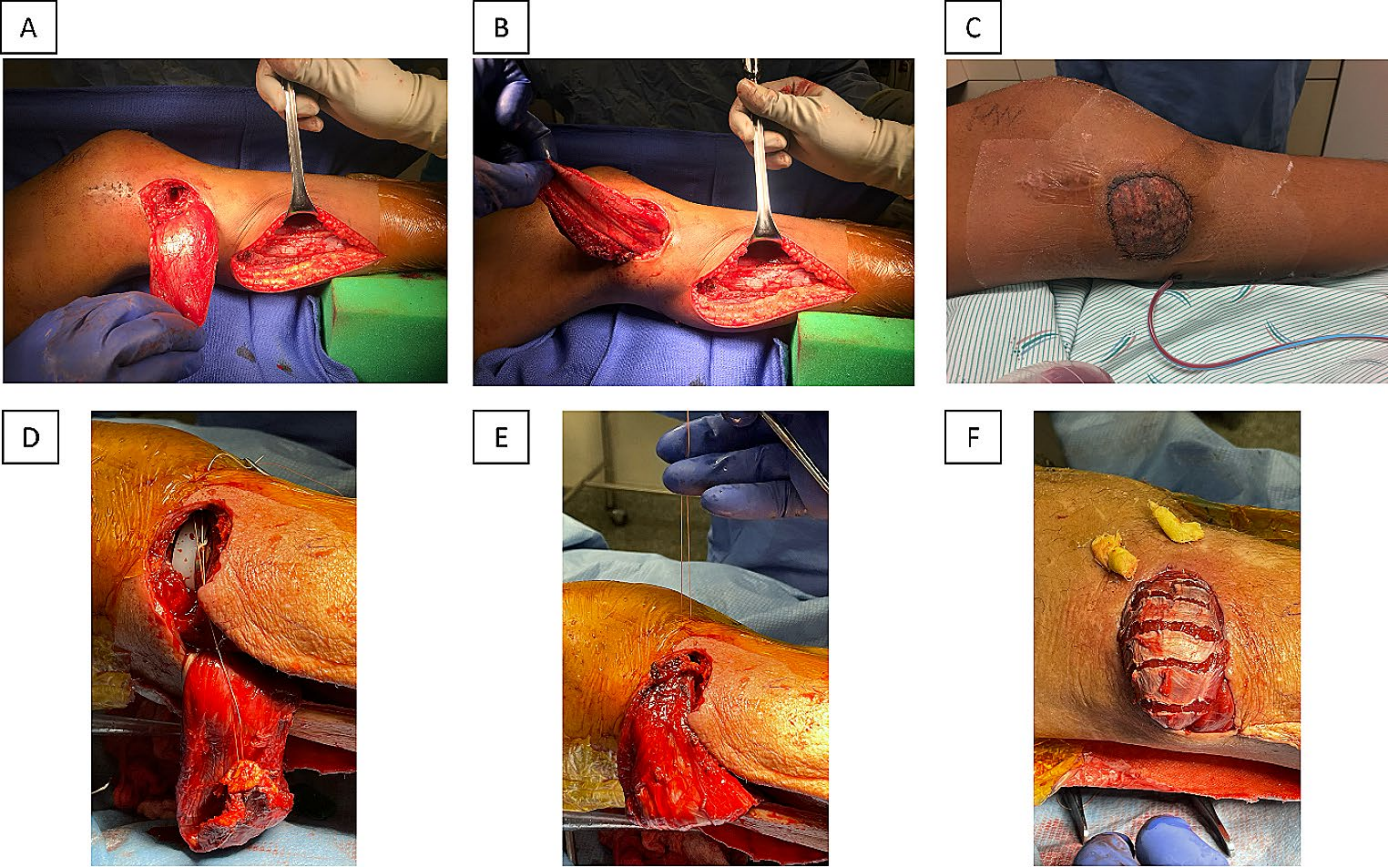
Technique for gastrocnemius flaps. **(A)**-**(C)** Lateral gastrocnemius flap. **(A)** Initial incision and harvesting the muscle and important anatomical relations. **(B)** Mobilization to cover the defect. **(C)** Closure. **(D)**-**(F)** Medial gastrocnemius flap. **(A)** Initial incision and harvesting the muscle and important anatomical relations. **(B)** Mobilization to cover the defect. **(C)** Closure

A medium sized soft tissue defect over the anterior aspect of the knee at or distal to the inferior pole of the patella was treated by using either a medial or lateral gastrocnemius flap (Fig. 2). The surgical procedure begins with the patient in the supine position with a tourniquet applied to the thigh. The location of the skin incision depends on whether a medial or lateral gastrocnemius flap is used. For the medial gastrocnemius flap, the incision starts 2 cm behind the posterior-medial border of the tibia and curves into the popliteal fossa. For the lateral flap, the incision is made on the lateral side. The skin and deep fascia are then opened, the muscle is dissected, and the neurovascular pedicle is exposed. The muscle is mobilized, transposed through a subcutaneous tunnel, and later covered with a split-thickness skin graft.

Intraoperative decision for a free latissimus dorsi flap was made in cases with larger defects proximal to the patella. It involves the transplantation of a section of the latissimus dorsi muscle, along with its overlying skin and blood vessels, from the back of the shoulder to the knee-defect. The surgical process necessitates precise microsurgical techniques to connect the blood vessels of the flap to those at the knee, ensuring adequate blood circulation for tissue viability.

Postoperatively, either a bolster dressing or a negative pressure was kept in place for 5 days with the knee in extension. Subsequently, the dressings were removed, and patients were allowed to bear weight. The knee was kept in extension for at least 2 weeks. Thereafter, a gradual range of motion was allowed as dictated by the procedure.

The average time to follow up or any kind of subsequent unplanned revision surgery was investigated and factors associated with revision surgery were analyzed. Furthermore, complication rates and potential risk factors for the respective wound coverage options were investigated.

In 44 (57.1%) cases, respective primary closures (*N* = 6) and flap procedures (*N* = 38) were performed to treat full-thickness soft tissue deficiency in the course of a periprosthetic joint infection (PJI). All of those patients fulfilled the Musculoskeletal Infection Society criteria (MSIS) for PJI [9]. Following the procedure, the patients received culture specific antimicrobials for treatment of their PJI for a minimum of six weeks. The infectious diseases consultant determined the choice of antibiotic regimen. The decision to continue oral antibiotics was based on adverse effects, the concern about recurrence of infection and drug tolerance. In contrast, in 33 (42.9%) cases, primary closures (*N* = 12) and flap procedures (*N* = 21) were performed to treat soft tissue defects after aseptic revision surgeries. Indications for aseptic revision surgeries are shown in Table 1.

**Table 1:** Revision indication and respective flap types

Flap Type Revision indication	Primary closure	Local fasciocutaneous flap	Medial gastrocnemius flap	Lateral gastrocnemius flap	Latissimus dorsi flap
Aseptic loosening / Instability	6	2	3	0	1
Periprosthetic fracture	2	3	1	0	0
Prolonged wound healing	4	3	2	0	0
Stiffness	0	1	1	0	1
Extensor mechanism deficiency	0	0	2	0	1
Periprosthetic joint infection	6	9	28	1	0
**Total**	**18**	**18**	**37**	**1**	**3**

To determine whether the timing of flap procedure plays a role, patients were divided into two groups: Group 1 - Flap coverage during a second stage procedure after eradicating the PJI (*N* = 9), and Group 2 - Flap procedure at the time of initial treatment for PJI including spacer insertion, polyethylene exchange or spacer exchange (*N* = 29). Planned revisions or second stage procedures were not counted as unplanned revision surgeries. The decision for the timing of the flap procedure was made by the plastic surgeon based on the respective soft tissue status during surgery.

The Shapiro-Wilk test was used to test normal distribution of the analyzed parameters. The Kaplan-Meier method was used to estimate cumulative revision-free survival. Estimated cumulative revision free survival rates at 5 years follow-up were reported. The log-rank test was used to compare the survival distributions of two samples. Univariate Cox regression analysis was used to assess the association of potential risk factors with the risk of revision surgery, which was reported as an unadjusted hazard ratio (HR) and 95% confidence interval (CI). Nonparametric independent variables were compared with Mann-Whitney tests. Statistical data analysis was performed with SPSS version 26 (IBM Corporation, New York, NY). The significance level was set at *P* ≤ 0.050.

## Results

### Patient characteristics

The study group consisted of 38 men and 39 women with a mean age of 62.6 (18.0–85.2) years and a mean BMI of 31.7 (20.1–54.2) kg/m² at the time of surgery. Furthermore, following comorbidities were recorded: diabetes mellitus (13 patients, 16.9%), peripheral vascular disease (9 patients, 11.7%) and current or former tobacco use (33 patients, 42.9%). The patient cohort had a mean of 3.8 (range 0–9) prior surgeries. The type of procedures and respective cohort characteristics are reported in Table 2. In 44 (57.1%) cases, respective procedures were performed to treat full-thickness soft tissue deficiency in the course of PJI. Table 3 shows the distribution of infectious organisms detected. In contrast, in 33 (42.9%) cases, primary closures (*N* = 12) and flap procedures (*N* = 21) were performed to treat soft tissue defects after aseptic revision surgeries.

**Table 2:** Knee wound closure types and patient characteristics

	Total number of patients		Age [years]		BMI [kg/m²]		Gender [male]		Prior Surgeries		Side [right]		Tabacco Use		Diabetes		PVD	
Group	*N*	%	Mean	SD	Mean	SD	*N*	%	Mean	SD	*N*	%	*N*	%	*N*	%	*N*	%
**Primary Closure**	18	23.4	60.8	12.3	31.6	5.3	9	50.0	3.9	2.2	10	55.6	6	33.3	0	0	1	5.6
**Medial gastrocnemius flap**	37	48.1	63.1	15.8	30.1	5,0	22	59.5	4.1	2.1	17	45.9	18	48.6	8	21.6	6	16.2
**Local fasciocutaneous flap**	18	23.4	63.9	12.5	35.9	8.3	6	33.3	3.3	2.9	9	50.0	7	38.9	4	22.2	2	11.1
**Free latissimus dorsi flap**	3	3.9	64.4	9.7	27.6	4.7	0	0	2.7	2.1	2	66.7	2	66.7	0	0	0	0
**Lateral gastrocnemius flap**	1	1.3	51.2		27.9		1	100	4		0	0	0	0	1	100	0	0
**Total**	77		62.6	13.8	31.7	6.4	38	49.4	3.8	2.3	38	49.4	33	42.9	13	16.9	9	11.7
***p-value***			0.666		0.071		0.125		0.431		0.779		0.610		0.040		0.754	

PVD = peripheral vascular disease; former or actual tabacco use

**Table 3:** Organisms Found in Patients with Positive Cultures

Organism	*N*
Polymicrobial	15
Staphylococcus	41
Staphylococcus aureus	15
Methicillin-resistant Staphylococcus aureus	12
Methicillin-resistant Staphylococcus epidermidis	7
Staphylococcus lugdunensis	3
Staphylococcus epidermidis	2
Coagulase-negative Staphylococcus	1
Staphylococcus capitis	1
Enterococcus	6
Enterobacter	4
Escherichia coli	4
Streptococcus viridans/agalactiae	2
Corynebacterium striatum	2
Candida albicans	2
Proteus mirabilis	1
Pseudomonas aeruginosa	1

### Low revision free survival rate of medial gastrocnemius flap procedures

**Fig. 3:**
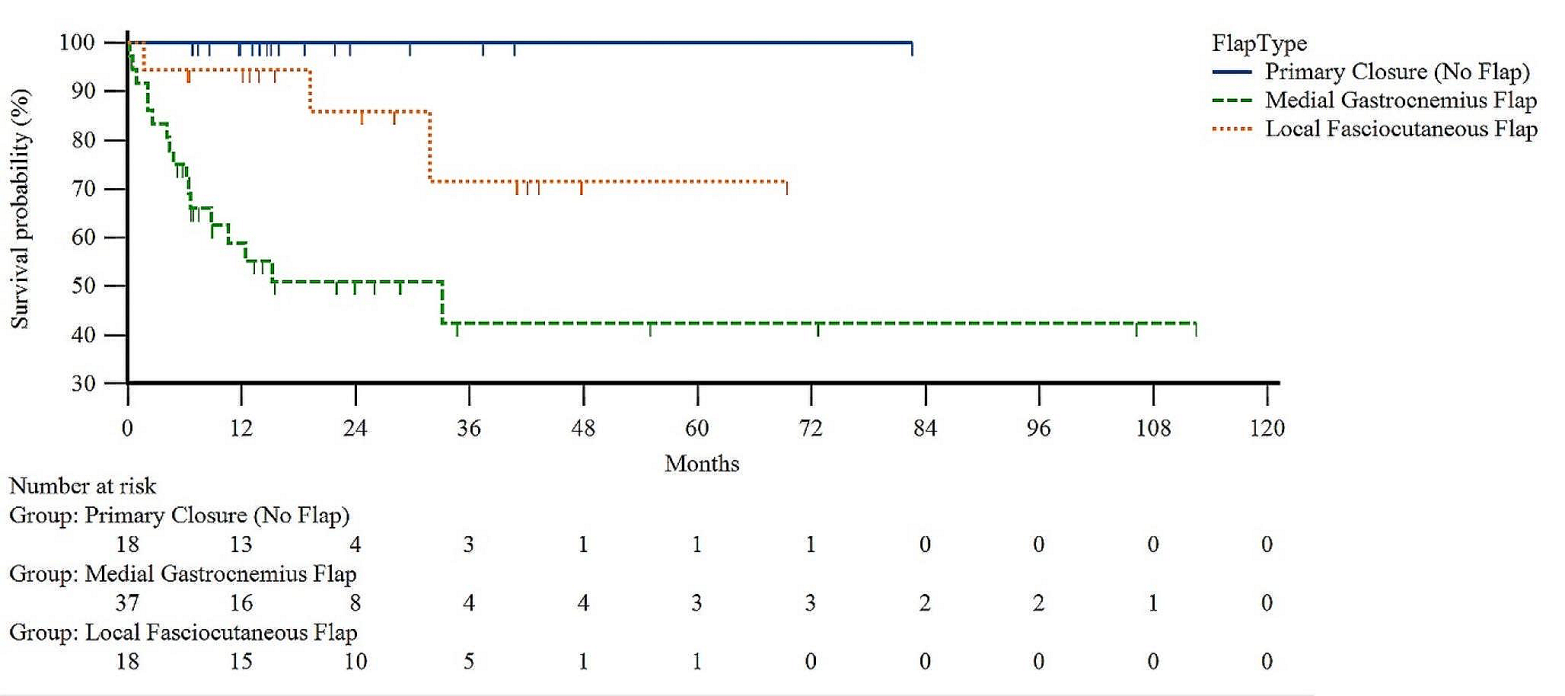
Cumulative revision-free survival rates of primary closures (blue), medial gastrocnemius (green) and local fasciocutaneous flaps (orange). The cumulative revision free survival rate of medial gastrocnemius flaps was significantly lower than the cumulative revision free survival rate of local fasciocutaneous flaps (*P* = 0.021) and primary closures (*P* > 0.001). Kaplan-Meier-Curve.

Cumulative revision free survival rates of primary closures (100%; *N* = 18) and total flap procedures (40.2%; *N* = 35) were significantly different (*p* = 0.005). Comparing cumulative revision free survival rates between the most common complex closure procedures in this patient cohort, the cumulative revision free survival rate of medial gastrocnemius flaps (42.5%; *N* = 20) was significantly lower than the cumulative revision free survival rate of local fasciocutaneous flaps (71.5%; *N* = 15; *P* = 0.021) and primary wound closures (100%; *N* = 18; *P* < 0.001). There was no significant difference between revision free survival rates of local fasciocutaneous flap procedures and primary wound closures (*P* = 0.156) (Fig. 3).

Medial gastrocnemius flaps had the highest prolonged wound healing (29.7%) rate, infection/reinfection rate (40.5%) and instability rate (13.5%). Complication rates and reasons for revision of the respective procedures are shown in Table 4. All of the free latissimus dorsi flaps (*N* = 3) and lateral gastrocnemius flap procedures (*N* = 1) underwent revision surgery.

**Table 4:** Knee wound closure types and respective complication rates

	Total number of patients		Any kind of revision surgery		Prolonged wound healing		Donor site compli-cation		Infection / Reinfection		Instab- ility		Arthrofibrosis / Stiffness		Aseptic loosening		Fracture		Arthrodesis / Amputation		Dea- th		Unplanned revision surgery
Group	*N*	%	*N*	%	*N*	%	*N*	%	*N*	%	*N*	%	*N*	%	*N*	%	*N*	%	*N*	%	*N*	%	Mean
**Primary Closure**	18	23.4	0	0.0	0	0.0	0	0.0	0	0.0	0	0.0	0	0.0	0	0.0	0	0.0	0	0.0	0	0.0	0.06
**Medial gastrocnemius flap**	37	48.1	17	45.9	11	29.7	1	2.7	15	40.5	5	13.5	0	0.0	0	0.0	1	2.7	3	8.1	2	5.4	1.03
**Local fasciocutaneous flap**	18	23.4	3	16.7	1	5.6	0	0.0	1	5.6	2	11.1	0	0.0	0	0.0	0	0.0	0	0.0	0	0.0	0.22
**Free latissimus dorsi flap**	3	3.9	3	100	0	0.0	0	0.0	1	33.3	0	0.0	2	66.7	1	33.3	1	33.3	0	0.0	0	0.0	1.33
**Lateral gastrocnemius flap**	1	1.3	1	100	0	0.0	0	0.0	1	100	0	0.0	0	0.0	0	0.0	0	0.0	0	0.0	0	0.0	1.0
**Total**	77		24	31.2	12	15.6	1	1.3	18	23.4	7	9.1	2	2.6	1	1.3	2	2.6	3	3.9	2	2.6	0.62

### Higher revision free survival rate of non-infection associated flap procedures

Comparing infection-associated (*N* = 38) and non-infection associated (*N* = 21) flap procedures, cumulative revision free survival rates were 30.5% (*N* = 19) versus 62.8% (*N* = 16; *P* = 0.047), respectively. In *N* = 22 of the 38 infection associated cases (57.9%), there was no recurrence of the PJI. Two (3.4%) initially aseptic revisions became infected perioperatively. There was one donor-site complication noted, which needed additional revision surgery (Table 4). The mean number of surgical procedures following the initial flap procedure was 1.08 (0–8) for infection-associated versus 0.29 (0–2) for non-infection-associated flap procedures.

### Timing of flap procedure during infection treatment

Knees with flaps performed during debridement with polyethylene exchange, spacer placement or spacer exchange (*N* = 29) had a trend towards a lower cumulative revision free survival rate (27.5%), compared to knees with flaps performed after successful treatment of PJI (*N* = 9) during second stage procedure (51.9%). This difference did not reach statistical significance (*p* = 0.127).

### Risk factors for revision of knee flap procedures

Risk factors for revision of knee flap procedures were analyzed using univariate Cox regression analysis (Table 5). Knees with more than two prior surgeries significantly increased the risk of revision (HR = 6.11, 95% CI = 2.34 to 15.95, *P* < 0.001). Furthermore, an age > 65 years significantly increased the risk of following revision surgeries (HR = 0.30, 95% CI = 0.11 to 0.81, *P* = 0.018).

**Table 5:** Unadjusted Univariate Cox Regression Analysis of Risk Factors for Revision and Failure of Knee Flap Procedures

		Revision	
Risk factor	*N*	HR (95% CI)	*P* value
*Infection*	38	2.62 (0.97–7.03)	0.056
*BMI > 40 kg/m²*	6	0.32 (0.04–2.34)	0.260
*Age > 65 years*	24	**0.30 (0.11–0.81)**	**0.018**
*Tabaco use*	27	1.46 (0.65–3.28)	0.354
*Diabetes*	13	1.13 (0.45–2.86)	0.796
*Peripheral Vascular Disease*	8	1.01 (0.30–3.44)	0.99
*Gender female*	30	1.36 (0.60–3.07)	0.459
*> 2 Prior surgeries*	6	**6.11 (2.34–15.95)**	**< 0.001**
*Resistant organism*	14	0.75 (0.28–1.97)	0.552

## Discussion

Wound complications during TKA are challenging, especially when they result in a vicious circle of wound breakdown and consequently compromised wound healing due to repeating operations and associated devascularization of the local soft tissue. To prevent this devastating course, a stable soft-tissue coverage of the wound is essential. A recent review of the current literature published by Chandra et al. showed that gastrocnemius flaps are most commonly used in challenging soft tissue defects in revision TKA (*N* = 421; 75.7%), followed by fasciocutaneous flaps (*N* = 78; 14%) and latissimus dorsi flaps (*N* = 41; 7.4%) [10]. The choice of the flap procedure mainly depends on the size of the defect, patients’ tolerance, and the experience of the surgeon. Most previous studies included patients with flap procedures performed by different surgeons [3, 5, 11, 12]. The experience of the surgeon plays an important role for the outcome of flap procedures, and therefore the inclusion of patients treated by different surgeons can add additional bias [13]. To avoid these confounding effects, the purpose of this study was to investigate the outcomes of a single, highly experienced plastic surgeon who regularly collaborates with the adult reconstruction team at the authors’ institution for soft tissue reconstruction of complex periprosthetic wounds in rTKA.

The current study reports a significantly higher revision free survival rate of primary closures compared to flap procedures, suggesting that whenever possible a primary wound closure should be preferred. One limitation of this study is that the exact defect sizes were not recorded at the time of surgery. Therefore, it is not possible to suggest a cut-off point which defect size should be closed primarily, and which defect size needs an additional flap procedure. Nevertheless, besides the defect size, the tension of the surrounding soft tissue, pre-existing scars and patients comorbidities also influence wound healing [6]. Houdek et al. showed an increased risk of amputation in patients with a wound size larger than 50 cm^2^ [11]. Therefore, future studies need to investigate the influence of defect size on respective flap outcomes in a prospective study with accurate intraoperative defect size measurements and documentation.

Furthermore, the results of this study suggest that fasciocutaneous flaps should be performed in small sized wounds to avoid the higher complication rate of muscular flaps. When comparing the outcomes of muscle and fasciocutaneous flaps previous studies showed comparable rates of complications, implant salvage and recurrence of infection [14–16]. In the case of deep infection, Corten et al. showed that 22/29 (92%) patients treated with gastrocnemius muscle flaps had satisfactory results at a mean follow-up of 4.5 years [17]. Hambardzumyan et al. described similar successful outcomes of gastrocnemius flap procedures (40/43; 93%) at a shorter mean follow-up of 18 months. In contrast, our data revealed a low cumulative revision free survival rate of flap procedures in patients with PJI (30.5%), compared to revision free survival rate of 62.8% in non-infection associated cases. Those markedly worse outcomes were also shown in other publications and may be caused by a higher case complexity (higher mean number of previous surgeries). In this context, Tetreault et al. elucidated that 14/27 patients who received a medial gastrocnemius flap for soft tissue coverage over an infected TKA had a persistent or recurrent infection, and that the overall survivorship of the prosthesis after flap procedure was low (48%) at 4 years [18]. Consistently, Kwiecien et al. showed that 58 patients who received a reactive flap treatment had a high rate of implant reinfection (58%), subsequent surgeries (2.2) and amputation (25%) at a mean follow-up of 67 months [5].

In addition, Kwiecien et al. showed that late reconstruction of soft tissue defects has a higher complication rate than early proactive flap coverage [5]. The authors compared patients with preexisting soft-tissue defects who required reactive flap procedures with patients who had no preexisting soft-tissue defects, but an expected extensive debridement during revision TKA requiring proactive flap coverage. Patients with no preexisting soft-tissue defects and a pro-active flap coverage had a significantly better outcome than patients with multiple prior revision surgeries and an obviously worse precondition. In this context, the current study identified more than two prior surgeries on the respective knee as a risk factor of revision (HR = 6.11, *P* < 0.001). Nevertheless, there was no significant difference between the cumulative revision free survival rate (27.5%) of knees with flaps performed during debridement with polyethylene exchange and knees with flaps performed after successful treatment of PJI during second stage procedure (51.9%). However, considering the significantly lower complication rate of non-infection associated flap closures, performing a flap procedure after successful treatment of PJI might be beneficial.

The current study has the following limitations: The data were collected retrospectively and its accuracy depended on adequate documentation at the time of service. This limited the collected data to what has been documented and may have caused selection bias. While a single plastic surgeon series limits the generalizability of the study results, this is also a strength of the study as there was a consistent approach to treating these defects and confounding effects and unobserved biases by multiple surgeons and different surgical techniques were prevented. Although the overall sample size was smaller compared to previous published studies, this is - to the best of our knowledge - the largest single plastic surgeon patient cohort described in the literature. The defect size was not documented at the time of surgery and therefore the study cannot give recommendations to when to consider flap procedures versus primary wound closure, however, the data suggest that all efforts should be made to obtain primary wound closure.

## Conclusion

In conclusion, the results of this study indicate that primary closure - if possible - should be preferred to early proactive flap coverage in reconstruction of complex periprosthetic wounds. Furthermore, fasciocutaneous flaps should be utilized in smaller defects to avoid the higher complication rate of muscular flaps. Even with the support of an experienced plastic surgeon who uses the same surgical technique, a high revision rate after initial flap procedure should be expected, regardless of the flap indication. Finally, the high revision rates of muscle flap closures in infection-associated and non-infection associated cases is an indication of the case complexity and not necessarily indicative for failure of the flap.

## Electronic supplementary material

Below is the link to the electronic supplementary material.

Supplementary Material 1

Supplementary Material 2

Supplementary Material 3

Supplementary Material 4

Supplementary Material 5

Supplementary Material 6
