# Supplementary material for: Complex periprosthetic wound coverage in patients undergoing revision total knee arthroplasty: a single plastic surgeon study
Source: Arch Orthop Trauma Surg. 2024 Apr 25;144(12):5093–100. doi: 10.1007/s00402-024-05240-6 (PMC11602818; doi:10.1007/s00402-024-05240-6)
Supplement: Supplementary file 2 — Supplementary Material 2 [file 402_2024_5240_MOESM2_ESM.pdf]

INDIVIDUAL CONFLICT OF INTEREST STATEMENT

American Association of Hip and Knee Surgeons

(Adopted from the American Academy of Orthopaedic Surgeons disclosure statement)

"Complex Periprosthetic Wound Coverage in Patients Undergoing Revision Total Knee Arthroplasty: A Single Plastic Surgeon Study."

Manuscript Title

1. Royalties from a company or supplier (The following conflicts were disclosed)

none

2. Speakers bureau/paid presentations for a company or supplier (The following conflicts were disclosed)

none

3A. Paid employee for a company or supplier (The following conflicts were disclosed)

none

3B. Paid consultant for a company or supplier (The following conflicts were disclosed)

none

3C. Unpaid consultants for a company or supplier (The following conflicts were disclosed)

none

4. Stock or stock options in a company or supplier (The following conflicts were disclosed)

none

5. Research support from a company or supplier as a Principal Investigator (The following conflicts were disclosed)

none

6. Other financial or material support from a company or supplier (The following conflicts were disclosed)

none

7. Royalties, financial or material support from publishers (The following conflicts were disclosed)

none

8. Medical/Orthopaedic publications editorial/governing board (The following conflicts were disclosed)

none

9. Board member/committee appointments for a society (The following conflicts were disclosed)

none

<u>Each author must sign AND print or type his/her name, date and submit a separate form</u>

In addition, one BLINDED Conflict of Interest form (no author names used) should be submitted per manuscript with all author disclosures.

Marco Brenneis	Marco Brenneis	23.01.2023
Author Name (Print or Type)	Author Signature	Date
