# Supplementary material for: Complex periprosthetic wound coverage in patients undergoing revision total knee arthroplasty: a single plastic surgeon study
Source: Arch Orthop Trauma Surg. 2024 Apr 25;144(12):5093–100. doi: 10.1007/s00402-024-05240-6 (PMC11602818; doi:10.1007/s00402-024-05240-6)
Supplement: Supplementary file 5 — Supplementary Material 5 [file 402_2024_5240_MOESM5_ESM.pdf]

INDIVIDUAL CONFLICT OF INTEREST STATEMENT

American Association of Hip and Knee Surgeons

(Adopted from the American Academy of Orthopaedic Surgeons disclosure statement)

The following form **must be filled out completely and submitted by each author (example, 6 authors, 6 forms). All items require a response. If there is no relevant disclosure for a given item, enter "*None*."**

"Complex Periprosthetic Wound Coverage in Patients Undergoing Revision Total Knee Arthroplasty: A Single Plastic Surgeon Study"

1. Royalties from a company or supplier (The following conflicts were disclosed)
Lima Corporate

2. Speakers bureau/paid presentations for a company or supplier (The following conflicts were disclosed)

Zimmer Biomet, Lima Corporate

3A. Paid employee for a company or supplier (The following conflicts were disclosed)
none

3B. Paid consultant for a company or supplier (The following conflicts were disclosed)
Depuy Synthes, Intellijoint Surgical, Lima Corporate, Zimmer Biomet

3C. Unpaid consultants for a company or supplier (The following conflicts were disclosed)
none

3. Stock or stock options in a company or supplier (The following conflicts were disclosed)
Intellijoint Surgical

4. Research support from a company or supplier as a Principal Investigator (The following conflicts were disclosed)
Zimmer Biomet, Intellijoint Surgical

5. Other financial or material support from a company or supplier (The following conflicts were disclosed)
none

6. Other financial or material support from a company or supplier (The following conflicts were disclosed)
none

7. Royalties, financial or material support from publishers (The following conflicts were disclosed)
none

8. Medical/Orthopaedic publications editorial/governing board (The following conflicts were disclosed)
None

9. Board member/committee appointments for a society (The following conflicts were disclosed)
none

Each author must sign AND print or type his/her name, date and submit a separate form

In addition, one BLINDED Conflict of Interest form (no author names used) should be submitted per manuscript with all author disclosures.

Peter Sculco	Peter Sculco	01/23/2023
Author Name (Print or Type)	Author Signature	Date
